# Validating Self-Reported Ad Recall as a Measure of Exposure to Digital Advertising: An Exploratory Analysis Using Ad Tracking Methodology

**DOI:** 10.3390/ijerph17072185

**Published:** 2020-03-25

**Authors:** Alexa R. Romberg, Morgane Bennett, Shreya Tulsiani, Bethany Simard, Jennifer M. Kreslake, Dionisios Favatas, Donna M. Vallone, Elizabeth C. Hair

**Affiliations:** 1 Schroeder Institute at Truth Initiative, Washington, DC 20001, USA; mbennett@truthinitiative.org (M.B.); stulsiani@truthinitiative.org (S.T.); bsimard@truthinitiative.org (B.S.); jkreslake@truthinitiative.org (J.M.K.); dvallone@truthinitiative.org (D.M.V.); ehair@truthinitiative.org (E.C.H.); 2 College of Global Public Health, New York University, New York City, NY 10012, USA; 3 Department of Prevention & Community Health, Milken Institute School of Public Health, George Washington University, Washington, DC 20052, USA; 4 Department of Health, Behavior and Society, Johns Hopkins Bloomberg School of Public Health, Baltimore, MD 21205, USA; 5 Marketing at Truth Initiative, Washington, DC 20001, USA; dfavatas@truthinitiative.org

**Keywords:** mass media, social marketing, campaign evaluation

## Abstract

Many mass media campaigns aimed at changing young people’s health behavior air on digital platforms rather than on broadcast media (e.g., television), given the intended audience’s preference for web-based communication. While research suggests self-reported ad recall correlates with exposure to television advertising, it remains unclear whether self-report measures are correlated with exposure to digital advertising. This study examined the association between an objective measure of digital ad exposure and self-reported recall of digital ads from the truth^®^ tobacco prevention campaign. Digital ad tracking methodology was employed to identify members of an online panel (ages 18−34) who had been exposed to ads during their regular web browsing. Demographics of exposed participants were used to develop a matched control group of non-exposed panel members. Members of the Exposed group (*n* = 458) and matched Control participants (*n* = 506) were surveyed on recall of truth ads, media use, and demographics. Results indicated that Exposed participants had significantly higher odds of reporting ad recall compared to Control participants. With each additional ad exposure, the odds of self-reporting higher frequency of ad exposure increased by 8% (OR = 1.08, 95% CI = 1.01−1.16). Findings suggest self-reported measures of ad recall are a valid measure of campaign exposure in a digital media environment.

## 1. Introduction

For several decades, mass media campaigns have contributed to changing health behavior [1,2]. Public health communication interventions are grounded in constructs found in multiple behavioral and systems theories [3]. Effective campaigns not only address individual factors (e.g., knowledge, attitudes, or beliefs) associated with health behavior but also work across the interpersonal, institutional, social, and environmental factors (e.g., social norms or structural inequities) that can facilitate or present barriers to enacting the targeted behavior [4,5,6]. Mass media campaigns have contributed to the decline in tobacco use in the United States, including national campaigns aimed at current smokers (Tips from Former Smokers from the Centers for Disease Control and Prevention (CDC)) and prevention campaigns aimed at adolescents (The Real Cost campaign from the Food and Drug Administration (FDA) and truth^®^ from Truth Initiative).

The impact of these anti-tobacco campaigns has been established by evaluation studies that examine the relationship between campaign exposure and changes in anti-tobacco knowledge, attitudes, beliefs, and tobacco use behavior [7,8,9,10,11,12,13,14,15]. Critical to the success of these campaigns is sufficient exposure to campaign messages in order demonstrate a population-level effect. In 2014, the CDC recommended reaching campaign awareness levels of at least 75% of the target audience quarterly with broadcast media (TV and radio) [16]. Although media message delivery has significantly changed, evaluation researchers continue to require valid measures of campaign exposure in order to assess campaign effectiveness.

Changes to the media landscape in recent years have introduced new challenges to measuring campaign exposure in order to evaluate campaign effectiveness. Media consumption has increasingly moved from broadcast media viewed on televisions to digital platforms, such as streaming services (e.g., Hulu), video hosting platforms (e.g., YouTube), internet websites, and social media (e.g., Instagram and Snapchat). This shift is most acute among adolescents and young adults, with the Pew Research Center reporting that 95% of teenagers have access to a smartphone [17,18]. Young adults aged 18–29 are regularly using social media platforms, with 77% of Snapchat users in this age group using the platform daily [19]. As a result, mass media campaigns have moved to digital media to more effectively reach these audiences.

Objective, exogenous measures of campaign exposure have been used in the evaluation of campaigns delivered exclusively or primarily through broadcast media (e.g., television and cable) [7,8,9]. Gross rating points (GRPs) are a population-level measure of the reach of an ad campaign and the frequency with which audience members are exposed to the messages [20]. Developed for broadcast media, GRPs are based on geographically defined media markets and assume uniformity in the population’s opportunity to view media content. GRPs are therefore not applicable to campaigns using digital media. 

Advertising for digital media, such as on streaming services, social media platforms, and internet websites, differs from traditional broadcast media advertising, in that, digital ads are displayed to audiences using algorithms that capitalize on user behavior and are not inherently bounded by geography [21]. Digital “impressions” are a metric of the number of digital ads delivered (i.e., loaded on a website or social media platform) for an advertising campaign. While digital impressions have been used as a proxy measure of digital message delivery, raw counts of impressions do not provide information on the size or characteristics of audiences potentially exposed to a digital ad, and therefore do not approximate ad reach. No standardized process has yet been developed to convert a raw count of impressions to a metric analogous to television GRPs. A recent evaluation of the U.S. Food and Drug Administration’s smoking-prevention campaign “The Real Cost” incorporated both television and digital Targeted Rating Points (i.e., TRPs, which are GRPs specific to the targeted audience) in their measure of campaign exposure [11]. However, a method of calculating digital TRPs was not established, and, the authors were unable to determine whether there was an independent effect of the digital ads on campaign outcomes.

Alternatively, subjective, endogenous measures of campaign exposure, such as self-reported ad recall, are also commonly used in campaign evaluation [12,13,14,15]. One strength of self-reported measures is that they assess exposure at the individual level, allowing for exposure and outcomes to be measured in the same population [22]. However, endogenous measures of exposure may be subject to recall bias and susceptible to confounding factors, such as selective attention, particularly among those engaging in the targeted health behaviors [23,24,25], and may also vary by platform and the environment in which the ad is viewed. For example, compared to ads viewed on television, digital ads are more likely to be viewed alone and within a more interactive environment [26]. It is not currently known how these differences between television and digital viewing experiences may influence self-reported measures of ad recall.

Self-reported measures of ad exposure have been found to correlate with objective measures of television campaign exposure as measured by GRPs [25,27,28,29] suggesting that self-report can be a valid measure of ad exposure. Given the lack of standardized, objective measures of digital media delivery, researchers have not been able to assess the validity of self-reported recall of ads viewed on digital platforms. Thus, validation of self-reported measures against objective metrics in a digital environment is a critical and necessary endeavor to documenting the impact of digital communication [22].

Digital technology now enables researchers to collect data on the number of digital ads that are shown to individual audience members. To do this, impressions are tagged with a digital signature, enabling each exposure during real-life web browsing to be logged for members of an opt-in online survey panel. Using cookies—a piece of software that records details about an individual’s interaction with a website—ad exposures among panel participants are tracked. This individual-level logging of digital campaign exposure offers opportunities to examine the validity of self-reported measures of digital campaign exposure [30].

Evaluations of the national anti-tobacco mass media campaign, truth^®^, have used both self-reported and objective (GRP) measures of campaign exposure to establish its effectiveness in changing tobacco-related knowledge, attitudes, and behaviors [7,13,15,31,32,33] However, the penetration and reach of digital communication platforms require a focused effort on examining how and to what extent traditional measures of exposure perform in this new landscape. The objective of this study was to utilize cookie technology to examine the association between a subjective measure of exposure to digital truth campaign ads (self-reported ad recall) and an objective measure of ad exposure (individual digital impressions). We hypothesized that (1) those with confirmed digital exposure to truth digital ads will be more likely to report ad recall compared to those not exposed, and (2) those exposed to a greater number of digital impressions will report having seen the ads more frequently than those exposed to fewer impressions. 

## 2. Materials and Methods

### 2.1. Participants

Participants were members of a national online, opt-in panel owned by Dynata. Panel participants aged 18−34 years were recruited on a rolling basis to form two between-subject groups: Digital Exposure, which included those who were exposed to at least one truth video ad on a digital platform (*n* = 458) and Control, which included participants who had not been digitally exposed (*n* = 506). Control participants were recruited from the Dynata panel to match Digital Exposure participants on gender, age, marital status, household income, and education. This study was reviewed by the Advarra Institutional Review Board.

### 2.2. Digital Exposure Measurement and Study Procedure

Data collection occurred on a rolling basis from 20 March 2019 to 15 July 2019. The ads used for this study were truth’s e-cigarette use-prevention videos, varying in length between 6, 15, and 30 s. The truth campaign employs a number of tactics to reach youth and young adults on digital platforms. While the objective of the campaign is to reach as many 15- to 24-year-olds as possible, ads may be targeted with higher frequency to specific audiences based on audience interests or geography (e.g., locations where smoking or vaping are more prevalent). In addition to audience-based targeting, ads are purchased programmatically, to appear with particular web content known to be popular with young adults, such as on YouTube, popular websites, or web-based episode players (e.g., online episodes on the CW network website or Sling).

Members of the panel shared their web-browsing activity with Dynata via cookies on their electronic devices. The first-party cookies used by Dynata provide a reliable record of web activity because they are not susceptible to clearing when a participant deletes cookies from their browser. The cookie logged information about truth ad impressions (i.e., timing, placement, and content of the ad displayed) with the panel participant ID each time a panel member encountered a truth digital video ad on internet websites in the course of their normal web browsing. Truth video ads also played on social networks such as Snapchat, Twitter, Instagram, and Facebook during the study period. However, the tracking system used was incompatible with these social networks and participants’ potential exposures to ads on social networks were not tracked or logged. 

Survey invitations were randomly sent to panelists within 24 h of a logged ad exposure and responses were required to be recorded within 48 h of exposure. The Digital Exposure group participants varied in the number of tracked ads to which they were exposed prior to receiving the survey invitation. After each Digital Exposure participant completed the survey, survey invitations were sent to matching unexposed panelists (i.e., panelists for whom no truth impressions had been logged) in order to recruit a Control participant that was otherwise similar to the Digital Exposure participant. Control participants were therefore panel members that were demographically similar to the Digital Exposure participants but who had not seen a tracked digital truth ad.

### 2.3. Measures

#### 2.3.1. Outcomes

Self-reported ad recall was assessed using a measure similar to those in other recent campaign evaluations [10,13,15]. Participants were presented a collage of screenshots from the ads aired during the fielding period and asked for a response to the following item: “These pictures are from multiple ads. Have you ever seen any of these ads?”. Ad recall was scored as a binary variable with 0 = no and 1 = yes.

Self-reported frequency of exposure was assessed among those who reported ad recall with the item “Overall, about how many times do you think you’ve seen these ads?” with response options “1−2 times,” “3−5 times,” and “more than 5 times.” Those who did not report recall were scored as “Never” for the frequency of exposure variable.

#### 2.3.2. Total Tracked Digital Video Impressions

As described above, each time a digital truth ad was displayed on a participant’s device, the details of the ad were logged. The total number of digital video impressions delivered to each participant was counted from the onset of the study through the date of their survey response. 

#### 2.3.3. Covariates

##### Potential Past-Week Television Exposure

In addition to the digital campaign, truth ads also ran on broadcast and cable television during the study period, including networks such as CW, MTV, and Comedy Central. To assess whether participants had the opportunity to see the ads on broadcast or cable television, the survey included questions about whether participants had watched television in the past 7 days on any of the networks on which truth bought advertising time. Participants were provided with a list of networks and, for each network they selected, participants indicated “the times they tuned in.” Options included time categories for weekday and weekends (e.g., primetime (weekday): Mon–Fri 8−11 p.m.). Based on the campaign’s weekly logs of ad placement, Dynata determined whether participants might have been exposed to the campaign on broadcast or cable television. A binary past-week TV “opportunity to see” measure (TV-OTS) was created, with a score of 1 for participants who reported watching a network at a time when a truth ad played according to weekly logs and a score of 0 for all others. As noted above, some participants may have encountered our ads while watching television content on web-based episode players. Exposure to ads within these players was tracked using the impression logging described above and was not part of the TV-OTS measure.

##### Demographic Characteristics

Demographic variables included were age, gender (male/female/another gender), race/ethnicity (non-Hispanic White/non-Hispanic Black/non-Hispanic Asian/Hispanic/non-Hispanic Other), household income (less than $25,000/$25,000–$50,000/more than $50,000), and current e-cigarette use (yes/no). E-cigarette use was measured with the yes-or-no question, “Have you ever tried using any e-cigarette/vape, including JUUL (even 1 or 2 puffs)?” accompanied by pictures of common e-cigarette products. Those who responded “Yes” were asked the follow-up question: “During the past 30 days, on how many days did you use an e-cigarette/vape, including JUUL (even 1 or 2 puffs)?” for which they could enter a number from 0 to 30. Those who reported use on one or more days in the past 30 days were coded as current users.

##### Weekly Media Use

Participants’ weekly television viewing was measured with the item, “In a typical week, how much time do you spend watching television?” with options “I do not watch television at all,” “Under 5 h,” “5−10 h,” “11−25 h,” and “more than 26 h.” Participants’ weekly social media use was measured with the item: “In a typical week, how much time do you spend on social media?” with the same response options.

### 2.4. Analysis Plan

Bivariate analyses used chi-square tests to assess differences between the exposure and control groups on covariates and binary ad recall. Logistic multiple regression was used to test for differences in binary ad recall between the Digital Exposure and Control groups. This model tested the hypothesis that participants with confirmed digital exposure to the ads were more likely to report ad recall than those without confirmed exposure.

A second model used ordered logistic regression models to test for a dose–response relationship between the four-level self-reported exposure frequency measure and total video impressions delivered. This model tested the hypothesis that, relative to participants exposed to fewer digital video ads, participants exposed to more ads were more likely to report having seen the ads a greater number of times.

The number of impressions across participants was highly positively skewed (see Results). The dose–response models were limited to participants in the Digital Exposure group (*n* = 404) who had 17 or fewer logged impressions (the 95th percentile of the impression count distribution) so that model estimates were not driven by the few participants (*n* = 21) with a larger number of impressions. A sensitivity analysis found the same patterns of significance when the full Digital Exposure group was included in the dose–response models.

Since participants were not randomly assigned to exposure groups, additional covariates that may be related to ad recall were also included in all models, including possible past-week television exposure to the campaign (TV-OTS), weekly television use, weekly social media use, current e-cigarette use, age, gender, race/ethnicity, and household income. There were 33 participants missing data on these covariates who were excluded from all models. Interactions between the digital exposure measure and TV-OTS were also tested for each model. However, no interactions were significant, and the non-significant interactions were removed from the model prior to the interpretation of effects.

## 3. Results

### 3.1. Participant Characteristics

Participant characteristics are presented in Table 1. Participants were, on average, 28 years old and more participants identified as female (73.2%) than as male (26.8%). The majority of participants reported a household income of >$50,000 (68.6%). As expected with the matched recruitment, the Digital Exposure and Control groups did not differ on demographic characteristics. However, relative to the Control group, a smaller proportion of participants in the Digital Exposure group were current e-cigarette users (17% (95% CI = 14.0–20.5) vs. 11.1% (95% CI = 8.6–14.4), respectively) and a smaller proportion had the opportunity to see the ads on TV in the past week (TV-OTS, 29.4% (95% CI = 25.6–33.6) vs. 23.1% (95% CI = 19.5–27.2)).

### 3.2. Exposure Group Comparisons

A larger percentage of participants in the Digital Exposure group than in the Control group reported ad recall (48.3% (95% CI = 43.7–52.8) vs. 29.6% (95% CI = 25.8–33.8), χ^2^ = 35.17, Table 1). This group difference was confirmed in the logistic model adjusting for covariates (Table 2). Those with confirmed Digital Exposure had over 250% higher odds of ad recall relative to Control participants (OR = 2.52, 95% CI = 1.87–3.40). Participants with potential past-week TV exposure (TV-OTS) also had higher odds of ad recall relative to those without (OR = 2.23, 95% CI = 1.59–3.12). There was no interaction between Digital Exposure and TV-OTS. Additionally, ad recall was significantly associated with current e-cigarette use (OR = 2.02, 95% CI = 1.32–3.10) and younger age (OR = 0.89, 95% CI = 0.85–0.92), but not with weekly TV or social media use or participant gender, race/ethnicity, or self-reported household income.

### 3.3. Dose–Response between Digital Exposure and Recall

Exposed participants varied in the number of digital impressions they had been exposed to prior to the survey. Total impressions ranged from 1 to 110. However, the distribution was highly skewed, with 45% of participants with one impression, 85% of participants with 5 or fewer impressions, and 95% (those included in the dose–response analyses) had 17 or fewer impressions.

**Figure 1:**
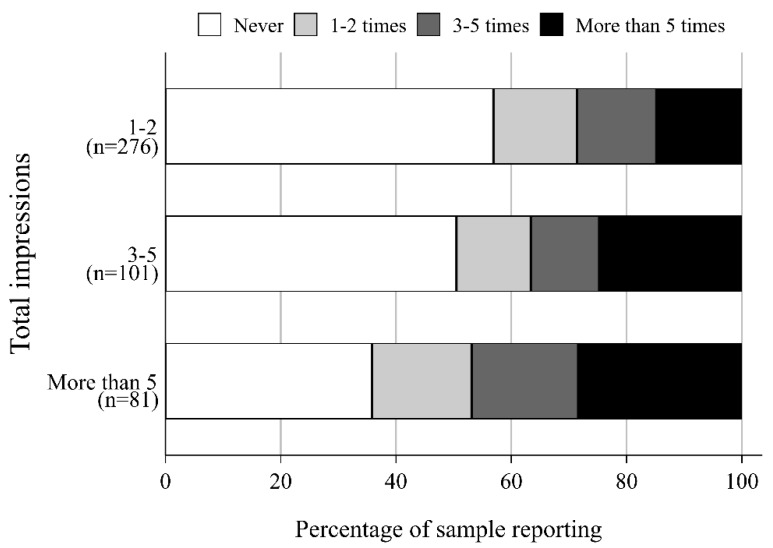
Observed self-reported frequency of exposure by the number of impressions delivered to participants in the Digital Exposure group (*n* = 404).

The relationship between total impressions and self-reported recall and frequency is shown in Figure 1, with impression totals grouped for ease of interpretation. Participants with more impressions were more likely to recall the ads and more likely to report having seen the ads 5 or more times.

Modeling results confirmed these patterns (Table 3). With each additional impression, the odds of self-reporting higher frequency of ad exposure relative to lower frequency increased by 8% (OR = 1.08, 95% CI = 1.01–1.16). Participants with TV-OTS also had higher odds of reporting higher ad frequency (OR = 1.84, 95% CI = 1.17–2.89), as did younger participants (OR = 0.91, 95% CI = 0.87–0.96). No other covariates were significantly related to self-reported ad frequency.

## 4. Discussion

This study provides evidence that confirmed digital ad exposure is significantly related to self-reported ad recall within a real-world digital context, even after controlling for possible exposure through other media platforms. Respondents with digital ad exposure were more likely to report ad recall compared with those who had not been exposed to digital ads, and those that received higher levels of ad exposure were more likely to report seeing ads more frequently than those who were delivered fewer ads. These findings suggest that participants’ recall of campaign ads is the result of, at least in part, exposure to ads through digital platforms.

To our knowledge, this is the first study to examine the relationship between self-reported ad recall and confirmed digital exposure. Prior research has identified mass media campaigns as some of the most effective behavior change interventions [1,2] and ensuring sufficient exposure of campaign messages among the target audience is a necessary component for determining campaign impact. While evaluation researchers have established valid measures for assessing campaign exposure to broadcast media, the same had not been established for digital media. Findings from the present study suggest that self-reported measures of ad recall can be used as valid measures of exposure to campaign content aired across digital platforms.

A particular strength of this study is the temporal contingency between the ad exposure and survey data collection, in that, respondents had to complete the data collection processes within 48 h after exposure. The tracking process of the panel members’ natural web browsing activity allowed for contact soon after digital ads were delivered. This method combines the ecological validity of a population-level ad recall study among respondents with varying levels of possible exposure with the rigor of a forced-exposure study in which ad exposure is guaranteed. 

This study is subject to limitations. First, those who participate in an opt-in commercial survey panel that tracks their web activity may differ from the general population. Second, the composition of the opt-in commercial panel used for the study was not representative of the overall target audience of the campaign. Most of the sample was female, which limits the generalizability of the findings. However, the gender distribution was consistent across the exposed and control groups. Third, exposed and non-exposed groups were not matched according to use of tobacco products, including e-cigarettes. Current tobacco product use has been found across multiple studies, including the results reported in this paper, to be associated with greater likelihood of self-reported recall of anti-tobacco ads [23,24,25]. A larger proportion of current e-cigarette users were in the control group (i.e., who were not exposed to digital ads but may have been exposed to television ads). As a result, the rate of ad recall in the control condition may reflect the higher prevalence of tobacco use in that group, and potentially underestimate the effect of digital exposure on ad recall. 

Despite our best efforts, the digital landscape does not allow researchers to monitor all digital properties for potential ad exposure. Social networks such as Snapchat and Facebook cannot be monitored using this ad tracking methodology. As a result, both the exposed and control participants may have viewed ads on those sites. To address this, our study included measures of TV viewing and social media use. However, these media measures may serve to underestimate the effect of the tracked digital ad exposure on ad recall. Future research may find larger differences between exposed and non-exposed groups.

## 5. Conclusions

As digital media platforms become central to the mass media landscape, health communication campaigns are increasingly using these digital platforms to efficiently deliver messages to their target audience. Given the challenges associated with objectively measuring exposure to digital content, such as restrictions put in place by social media companies and users’ concerns about privacy, researchers continue to rely on self-reported measures to estimate exposure to ad campaigns [34]. Our results indicate that self-reported ad recall provides a meaningful measure of exposure to public health messages delivered on digital platforms. Further research is needed to assess the validity of self-reported recall measures for digital campaign exposure among young people who are often the target of health promotion education efforts. Additionally, further research could assess the validity of self-reported recall measures with variations in question wording and content (e.g., aided recall vs. unaided). Given the rapid shift from broadcast to digital media use among young people, future efforts need to focus on developing and testing measures of campaign exposure in a controlled digital environment.

## Figures and Tables

**Table 1:** Sample characteristics, frequencies (column percentages), or means (SD).

	Total	Exposure Groups		
		Control	Digital	*p*-value
	*n* = 964	*n* = 506	*n* = 458	
**Age**				
Mean (SD)	28.3 (0.14)	28.4 (0.19)	28.1 (0.20)	0.329
**Gender Identity**				
Female	697 (73.2)	357 (71.7)	340 (74.9)	0.265
Male	255 (26.8)	141 (28.3)	114 (25.1)	
**Race/Ethnicity**				
NH-White	632 (65.8)	332 (66.1)	300 (65.5)	0.958
NH-Black	82 (8.5)	45 (9.0)	37 (8.1)	
NH-Asian	98 (10.2)	49 (9.8)	49 (10.7)	
Hispanic	107 (11.1)	56 (11.2)	51 (11.1)	
NH-Other	41 (4.3)	20 (4.0)	21 (4.6)	
**Household Income**				
<$25K	104 (11.5)	48 (10.0)	56 (13.1)	0.241
$25−50K	181 (20.0)	92 (19.2)	89 (20.8)	
>$50K	622 (68.6)	339 (70.8)	283 (66.1)	
**Current E-Cigarette Use**				
No	827 (85.8)	420 (83.0)	407 (88.9)	0.009
Yes	137 (14.2)	86 (17.0)	51 (11.1)	
**TV-OTS**				
No	709 (73.5)	357 (70.6)	352 (76.9)	0.027
Yes	255 (26.5)	149 (29.4)	106 (23.1)	
**Weekly TV**				
No use	24 (2.5)	12 (2.4)	12 (2.6)	0.759
<5 h	158 (16.4)	77 (15.2)	81 (17.7)	
5−10 h	310 (32.2)	163 (32.2)	147 (32.1)	
11−25 h	350 (36.3)	192 (37.9)	158 (34.5)	
26+ h	122 (12.7)	62 (12.3)	60 (13.1)	
**Weekly Social Media**				
No use	38 (3.9)	22 (4.3)	16 (3.5)	0.858
<5 h	208 (21.6)	113 (22.3)	95 (20.7)	
5−10 h	335 (34.8)	170 (33.6)	165 (36.0)	
11−25 h	261 (27.1)	135 (26.7)	126 (27.5)	
26+ h	122 (12.7)	66 (13.0)	56 (12.2)	
**Ad Recall**				
No	593 (61.5)	356 (70.4)	237 (51.8)	<0.001
Yes	371 (38.5)	150 (29.6)	221 (48.3)	

Note: *p*-values represent χ^2^ tests for differences between exposure groups. Some frequencies do not sum to the sample total because of missing data. NH = non-Hispanic; TV-OTS = television, opportunity to see.

**Table 2:** Logistic model results predicting binary ad recall.

	OR	95% CI
**Exposure**		
Control	Ref	
Digital	2.52 ***	1.87–3.40
**TV-OTS**		
No	Ref	
Yes	2.23 ***	1.59–3.12
**Weekly TV**		
No use	0.69	0.23–2.09
<5 h	Ref	
5−10 h	1.02	0.64–1.62
11−25 h	1.44	0.91–2.30
26+ h	1.14	0.64–2.03
**Weekly Social Media**		
No use	0.82	0.34–1.97
<5 h	Ref	
5−10 h	1.14	0.76–1.72
11−25 h	0.80	0.52–1.25
26+ h	0.98	0.57–1.70
**Current E-Cigarette Use**		
No	Ref	
Yes	2.02 **	1.32–3.10
**Age**		
Years	0.89 ***	0.85–0.92
**Gender**		
Female	Ref	
Male	1.02	0.73–1.43
**Race/Ethnicity**		
NH-White	Ref	
Hispanic	1.50	0.94–2.38
NH-Black	0.80	0.46–1.38
NH-Asian	0.73	0.44–1.23
NH-Other	1.01	0.47–2.20
**Household Income**		
<$25K	1.34	0.83–2.18
$25−50K	1.38	0.95–2.00
>$50K	Ref	

OR = odds ratio, CI = confidence interval; ** *p* < 0.01, *** *p* < 0.001.

**Table 3:** Ordered logistic model output predicting self-reported frequency of exposure.

	OR	95% CI
**Digital Exposure**		
Total Impressions	1.08 *	1.01–1.16
**TV-OTS**		
No	Ref	
Yes	1.84 **	1.17–2.89
**Weekly TV**		
No use	0.72	0.18–2.81
<5 h	Ref	
5−10 h	1.35	0.75–2.44
11−25 h	1.70	0.94–3.07
>26 h	1.91	0.90–4.05
**Weekly Social Media**		
No use	0.78	0.21–2.91
<5 h	Ref	
5−10 h	1.04	0.61–1.77
11−25 h	0.66	0.37–1.21
26+ h	0.90	0.43–1.88
**Current E-Cigarette Use**		
No	Ref	
Yes	0.95	0.51–1.77
**Age**		
Years	0.91 ***	0.87–0.96
**Gender**		
Female	Ref	
Male	0.76	0.48–1.98
**Race/Ethnicity**		
NH-White	Ref	
Hispanic	1.07	0.60–1.91
NH-Black	0.93	0.44–1.98
NH-Asian	0.92	0.48–1.76
NH-Other	2.44	0.96–6.22
**Household Income**		
<$25K	1.22	0.66–2.27
$25−50K	1.45	0.90–2.34
>$50K	Ref	

OR = odds ratio, 95% CI = 95% confidence interval; * *p* < 0.05, ** *p* < 0.01, *** *p* < 0.001.

## References

[1] Allen J.A., Duke J.C., Davis K.C., Kim A.E., Nonnemaker J.M., Farrelly M.C. (2015). Using mass media campaigns to reduce youth tobacco use: A review. Am. J. Health Promot..

[2] Wakefield M.A., Loken B., Hornik R.C. (2010). Use of mass media campaigns to change health behaviour. Lancet.

[3] Maibach E., Parrott R.L. (1995). Designing Health Messages: Approaches from Communication Theory and Public Health Practice.

[4] Abroms L.C., Maibach E.W. (2008). The Effectiveness of Mass Communication to Change Public Behavior. Annu. Rev. Public Health.

[5] Moran M.B., Frank L.B., Zhao N., Gonzalez C., Thainiyom P., Murphy S.T., Ball-Rokeach S.J. (2016). An Argument for Ecological Research and Intervention in Health Communication. J. Health Commun..

[6] Richard L., Gauvin L., Raine K. (2011). Ecological Models Revisited: Their Uses and Evolution in Health Promotion Over Two Decades. Annu. Rev. Public Health.

[7] Farrelly M.C., Nonnemaker J., Davis K.C., Hussin A. (2009). The influence of the national truth^®^ campaign on smoking initiation. Am. J. Prev. Med..

[8] Duke J.C., Farrelly M.C., Alexander T.N., MacMonegle A.J., Zhao X., Allen J.A., Delahanty J.C., Rao P., Nonnemaker J. (2018). Effect of a national tobacco public education campaign on youth’s risk perceptions and beliefs about smoking. Am. J. Health Promot..

[9] Kranzler E.C., Hornik R.C. (2019). The Relationship Between Exogenous Exposure to “The Real Cost” Anti-smoking Campaign and Campaign-Targeted Beliefs. J. Health Commun..

[10] McAfee T., Davis K.C., Alexander R.L., Pechacek T.F., Bunnell R. (2013). Effect of the first federally funded US antismoking national media campaign. Lancet.

[11] Duke J.C., MacMonegle A.J., Nonnemaker J.M., Farrelly M.C., Delahanty J.C., Zhao X., Smith A.A., Rao P., Allen J.A. (2019). Impact of The Real Cost Media Campaign on Youth Smoking Initiation. Am. J. Prev. Med..

[12] Duke J.C., Alexander T.N., Zhao X., Delahanty J.C., Allen J.A., MacMonegle A.J., Farrelly M.C. (2015). Youth’s awareness of and reactions to The Real Cost national tobacco public education campaign. PLoS ONE.

[13] Hair E.C., Cantrell J., Pitzer L., Bennett M.A., Romberg A.R., Xiao H., Rath J.M., Halenar M.J., Vallone D. (2018). Estimating the pathways of an antitobacco campaign. J. Adolesc. Health.

[14] Farrelly M.C., Duke J.C., Nonnemaker J., MacMonegle A.J., Alexander T.N., Zhao X., Delahanty J.C., Rao P., Allen J.A. (2017). Association between The Real Cost media campaign and smoking initiation among youths—United States, 2014–2016. MMWR Morb. Mortal. Wkly. Rep..

[15] Vallone D., Cantrell J., Bennett M., Smith A., Rath J.M., Xiao H., Greenberg M., Hair E.C. (2017). Evidence of the Impact of the truth FinishIt Campaign. Nicotine Tob. Res..

[16] Centers for Disease Control and Prevention (2014). Best Practices for Comprehensive Tobacco Control Programs—2014.

[17] Pew Research Center (2018). Teens, Social Media, and Technology 2018.

[18] Villanti A.C., Johnson A.L., Ilakkuvan V., Jacobs M.A., Graham A.L., Rath J.M. (2017). Social media use and access to digital technology in US young adults in 2016. J. Med. Internet Res..

[19] Pew Research Center (2019). Share of U.S. Adults Using Social Media, Including Facebook, Is Mostly Unchanged Since 2018.

[20] Farris P.W., Bendle N., Pfeifer P., Reibstein D. (2010). Marketing Metrics: The definitive Guide to Measuring Marketing Performance.

[21] Evans W.D., Thomas C.N., Favatas D., Smyser J., Briggs J. (2019). Digital Segmentation of Priority Populations in Public Health. Health Educ. Behav..

[22] Niederdeppe J. (2014). Conceptual, empirical, and practical issues in developing valid measures of public communication campaign exposure. Commun. Methods Meas..

[23] Biener L., McCallum-Keeler G., Nyman A.L. (2000). Adults’ response to Massachusetts anti-tobacco television advertisements: Impact of viewer and advertisement characteristics. Tob. Control.

[24] Slater M.D. (2004). Operationalizing and analyzing exposure: The foundation of media effects research. J. Mass Commun. Q..

[25] Cowling D.W., Modayil M.V., Stevens C. (2010). Assessing the relationship between ad volume and awareness of a tobacco education media campaign. Tob. Control..

[26] Loughney M., Eichholz M., Hagger M. (2008). Exploring the effectiveness of advertising in the ABC.com full episode player. J. Advert. Res..

[27] Biener L., Wakefield M., Shiner C.M., Siegel M. (2008). How broadcast volume and emotional content affect youth recall of anti-tobacco advertising. Am. J. Prev. Med..

[28] Dunlop S., Perez D., Cotter T. (2012). The natural history of antismoking advertising recall: The influence of broadcasting parameters, emotional intensity and executional features. Tob. Control.

[29] Niederdeppe J. (2005). Assessing the validity of confirmed ad recall measures for public health communication campaign evaluation. J. Health Commun..

[30] Flosi S., Fulgoni G., Vollman A. (2013). If an Advertisement Runs Online And No One Sees It, Is It Still an Ad?: Empirical Generalizations in Digital Advertising. J. Advert. Res..

[31] Farrelly M.C., Davis K.C., Duke J., Messeri P. (2008). Sustaining ‘truth’: Changes in youth tobacco attitudes and smoking intentions after 3 years of a national antismoking campaign. Health Educ. Res..

[32] Farrelly M.C., Davis K.C., Haviland M.L., Messeri P., Healton C.G. (2005). Evidence of a dose-response relationship between “truth” antismoking ads and youth smoking prevalence. Am. J. Public Health.

[33] Vallone D., Greenberg M., Xiao H., Bennett M., Cantrell J., Rath J., Hair E. (2017). The effect of branding to promote healthy behavior: Reducing tobacco use among youth and young adults. Int. J. Environ. Res. Public Health.

[34] Jerit J., Barabas J., Pollock W., Banducci S., Stevens D., Schoonvelde M. (2016). Manipulated vs. Measured: Using an Experimental Benchmark to Investigate the Performance of Self-Reported Media Exposure. Commun. Methods Meas..

